# Consequences of phosphoenolpyruvate:sugar phosphotranferase system and pyruvate kinase isozymes inactivation in central carbon metabolism flux distribution in *Escherichia coli*

**DOI:** 10.1186/1475-2859-11-127

**Published:** 2012-09-13

**Authors:** Eugenio Meza, Judith Becker, Francisco Bolivar, Guillermo Gosset, Christoph Wittmann

**Affiliations:** 1 Cellular Engineering and Biocatalysis Department, Biotechnology Institute, Universidad Nacional Autónoma de México, Apdo. Postal 510-3, Cuernavaca, Morelos, 62210, Mexico; 2 Institute of Biochemical Engineering, Technische Universität Braunschweig, Gaußstr. 17, D-38106, Braunschweig, Germany

**Keywords:** PTS, Pyruvate kinase, Aromatic amino acids

## Abstract

**Background:**

In *Escherichia coli* phosphoenolpyruvate (PEP) is a key central metabolism intermediate that participates in glucose transport, as precursor in several biosynthetic pathways and it is involved in allosteric regulation of glycolytic enzymes. In this work we generated W3110 derivative strains that lack the main PEP consumers PEP:sugar phosphotransferase system (PTS^-^) and pyruvate kinase isozymes PykA and PykF (PTS^-^*pykA*^-^ and PTS^-^*pykF*^-^). To characterize the effects of these modifications on cell physiology, carbon flux distribution and aromatics production capacity were determined.

**Results:**

When compared to reference strain W3110, strain VH33 (PTS^-^) displayed lower specific rates for growth, glucose consumption and acetate production as well as a higher biomass yield from glucose. These phenotypic effects were even more pronounced by the additional inactivation of PykA or PykF. Carbon flux analysis revealed that PTS inactivation causes a redirection of metabolic flux towards biomass formation. A cycle involving PEP carboxylase (Ppc) and PEP carboxykinase (Pck) was detected in all strains. In strains W3110, VH33 (PTS^-^) and VH35 (PTS^-^, *pykF*^-^), the net flux in this cycle was inversely correlated with the specific rate of glucose consumption and inactivation of Pck in these strains caused a reduction in growth rate. In the PTS^-^ background, inactivation of PykA caused a reduction in Ppc and Pck cycling as well as a reduction in flux to TCA, whereas inactivation of PykF caused an increase in anaplerotic flux from PEP to OAA and an increased flux to TCA. The wild-type and mutant strains were modified to overproduce L-phenylalanine. In resting cells experiments, compared to reference strain, a 10, 4 and 7-fold higher aromatics yields from glucose were observed as consequence of PTS, PTS PykA and PTS PykF inactivation.

**Conclusions:**

Metabolic flux analysis performed on strains lacking the main activities generating pyruvate from PEP revealed the high degree of flexibility to perturbations of the central metabolic network in *E. coli*. The observed responses to reduced glucose uptake and PEP to pyruvate rate of conversion caused by PTS, PykA and PykF inactivation included flux rerouting in several central metabolism nodes towards anabolic biosynthetic reactions, thus compensating for carbon limitation in these mutant strains. The detected cycle involving Ppc and Pck was found to be required for maintaining the specific growth and glucose consumption rates in all studied strains. Strains VH33 (PTS^-^), VH34 (PTS^-^*pykA*^-^) and VH35 (PTS^-^*pykF*^-^) have useful properties for biotechnological processes, such as increased PEP availability and high biomass yields from glucose, making them useful for the production of aromatic compounds or recombinant proteins.

## Background

**Figure 1:**
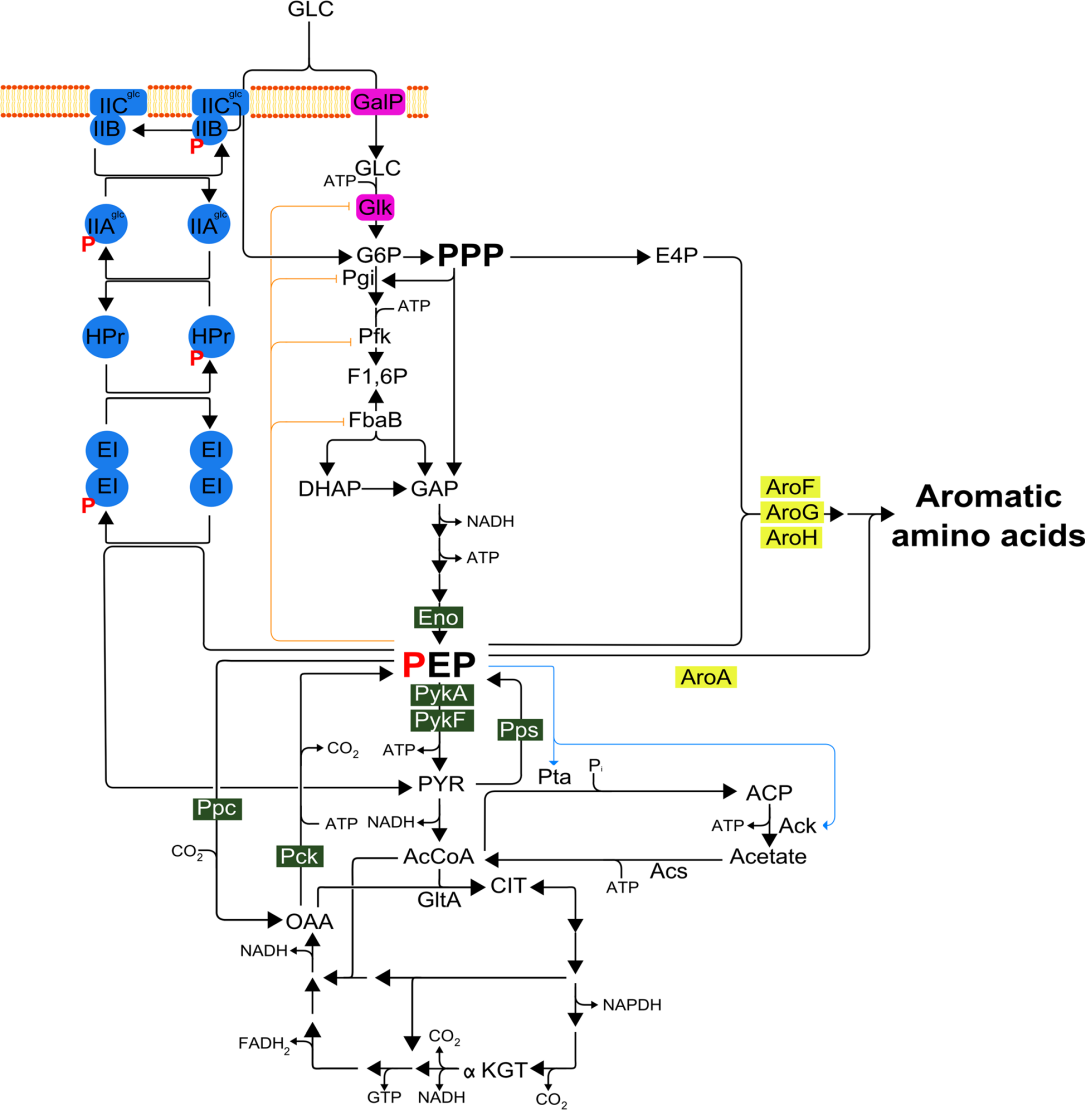
**Central carbon metabolism pathways in *E. coli.*** The Embden-Meyerhof-Parnas (EMP), pentose phosphate pathway (PPP) and tricarboxylic acid cycle (TCA) in *E.* coli during growth in glucose. In the figure PTS (in blue), the non PTS glucose uptake system (in purple), PEP node (green) and AAA (yellow) enzymes are depicted. The allosteric effects that PEP has in EMP and acetate biosynthesis are in orange line for inhibition and blue arrow for activation.

*Escherichia coli* is a fast growing microorganism that can be modified by a wide variety of molecular tools, thus enabling the generation of industrial strains for the production of metabolites and recombinant proteins. This bacterium can grow in defined media using glucose as sole carbon source, and under this condition, central metabolism is mainly constituted by the Embden-Meyerhof-Parnas (EMP) pathway, the pentose phosphate pathway (PPP) and the tricarboxylic acid (TCA) cycle. Together, these three pathways provide energy (ATP, GTP), reducing power (NADH, NADPH, FADH_2_) and biomass precursors for the cell [1]. When *E. coli* grows on glucose as carbon source, this sugar is imported by the phosphoenolpyruvate (PEP):sugar phosphotransferase system (PTS) that couples the transfer of the phosphate group from PEP to this sugar, thus generating as products glucose 6-phosphate (G6P) and pyruvate (PYR) [2-4] (Figure 1). In *E. coli*, PEP also participates as substrate in the reaction catalyzed by the pyruvate kinase (Pyk) isozymes (PykA and PykF), yielding PYR and ATP [5]. Furthermore, PEP connects the EMP pathway and the TCA cycle via an anaplerotic reaction catalyzed by PEP carboxylase (Ppc) that generates oxaloacetate (OAA) [6,7]. During gluconeogenic metabolism PEP synthase (Pps) converts PYR to PEP and PEP carboxykinase (Pck) forms PEP from OAA [7,8]. In addition to its participation in metabolic reactions, PEP also plays a regulatory role. An inhibitory effect of PEP has been described on the EMP pathway enzymes glucokinase (Glk), phosphoglucoisomerase (Pgi), phosphofructokinase (Pfk) and aldolase (FbaA) [9] whereas an activation effect has been described in the enzymes phosphate acetyltransferase (Pta) and acetate kinase (Aks) [10] (Figure 1). 

**Figure 2:**
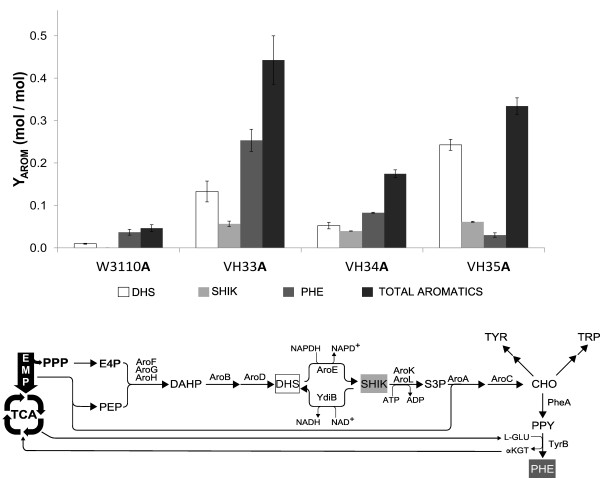
**Aromatic amino acids biosynthesis. a**) DHS, SHIK, PHE and (DHS + SHIK + PHE) yields from glucose. The values shown are the result of three independent experiments. **b**) The common aromatic pathway and the final pathways leading to PHE, TYR and TRP.

The first step in the common aromatic pathway or shikimate (SHIK) pathway consists in the condensation of PEP and erythrose 4 phosphate (E4P) to yield 3-deoxy-D-*arabino*heptulosonate 7 phosphate (DAHP). In *E. coli*, this reaction is catalyzed by the DAHP synthase isozymes (AroF, AroG and AroH) [11] (Figures 1 and 2b). Within this pathway, the synthesis of dehydroshikimate (DHS) to yield SHIK, a reaction catalyzed by the enzyme SHIK dehydrogenase (AroE), is a step that requires reducing power in the form of NADPH. An additional PEP molecule is required to condense with SHIK-3-phosphate (S3P) to yield 5-enol-pyruvylshikimate-3-phosphate (EPSP) by the enzyme EPSP synthase (AroA) [12]. The final product of the SHIK pathway is chorismate (CHO), a precursor molecule for the synthesis of the aromatic amino acids tryptophan (TRP), tyrosine (TYR) and phenylalanine (PHE) [13] (Figure 2b). The PHE biosynthetic pathway starts with the conversion of CHO to prephenate and subsequently to phenylpyruvate (PPY) by the bifunctional enzyme CHO mutase-prephenate dehydratase (CM-PDT), encoded by gene *pheA*. In the last step of this pathway, the enzyme PHE aminotransferase (TyrB) transfers an amino group from glutamate to PPY to yield PHE (Figure 2b). 

The aromatic amino acids and some intermediates of the SHIK pathway are metabolites having industrial applications [14-19]. These include the aromatic amino acid PHE as precursor of the low-calorie sweetener aspartame, L-3,4-dihydroxyphenylalanine (L-DOPA) used for treatment of Parkinson's disease [20] and the intermediate SHIK as precursor of the antiviral osetalmivir [21,22]. Several reports indicate that PEP availability is one of the main factors to be considered when designing a strategy to generate aromatics production strains [19,23,24]. In this regard, PTS, PykA and PykF, as the main PEP consuming reactions in *E. coli*, have been the subject of several studies to understand their role on the cell’s physiology [25-29]. In *E. coli*, PTS is the main PEP-consuming activity during growth on glucose [2-4]. For this reason, PTS can be considered the most promising cellular target to inactivate in order to increase PEP availability and therefore, aromatics yield from glucose [15]. A mutant strain lacking PTS activity (PTS^-^) displays poor growth on glucose (PTS^-^ glc^-^ phenotype) which is clearly an undesirable trait in the context of an industrial production process. To improve the characteristics of a PTS^-^ glc^-^ strain, a chemostat selection method was employed, thereby it was possible to isolate mutants that display rapid growth on glucose (PTS^-^ glc^+^ phenotype) [30]. Characterization of one of such mutants revealed that glucose import and phosphorylation are dependent on the GalP symporter and glucokinase (Glk), respectively [27,31] (Figure 1). This mutant was modified for aromatics production and it was determined that it can produce PHE with a higher yield from glucose when compared to an isogenic PTS^+^ strain [23,30,32].

In *E. coli* the total Pyk activity is dependent on the PykA and PykF isozymes which constitute the second main PEP-consuming activity in the cell [5,33-35]. These isozymes are encoded by genes *pykA* and *pykF*, whereby *pykF* is regulated by the catabolite repressor/activator protein (Cra) [36,37]. It has recently been demonstrated that *pykA* might also be regulated by Cra [38]. Both Pyk isozymes are subject to metabolic control of enzyme activity. The enzyme PykF is activated by fructose-1, 6-bisphosphate (FBP), whereas ATP, GTP and succinyl coenzyme A (SUCC CoA) are negative effectors [36,39-44]. The enzyme PykA is activated by adenosine monophosphate (AMP) and sugars from the PPP (gluconate 6-phosphate, ribose 5-phosphate, ribu-lose 5-phosphate, heptulosonate 7-phosphate and xylulose 5-phosphate) whereas SUCC CoA is a negative effector [45,46]. It has been observed in *E. coli* that PykF displays the highest enzyme activity when compared to PykA in wild type as well as the PTS^-^ background during growth on glucose as sole carbon source [28]. Based on these data, PykF is considered the main Pyk activity in *E*. *coli*[25,26,29].

Both PykA and PykF are well characterized proteins, however, their specific roles in central carbon metabolism are still not completely clear. Carbon flux studies in Pyk deficient *E. coli* strains have been performed in a genetic background where PTS is active, therefore, PTS-dependent synthesis of PYR from PEP is still occurring by this activity in the mutant strains [25,26,29]. An *E. coli* strain lacking PTS activity provides a background where the PEP to PYR reaction is dependent only on Pyk activity, thus enabling a study designed to provide further insight into the roles of PTS, PykA and PykF on central metabolism. The use of several “omics” tools to unravel *E*. *coli*'s cellular responses has allowed a better understanding of the physiology of this important industrial microorganism [25,26,29]. In this work we used a fluxome [47] approach to describe the phenotypic response (carbon fluxes) to the absence of PTS and also to a condition when the PYR to PEP reaction is dependent only on PykA or PykF activity.

## Methods

### Strains and plasmids

The strains used in this work are described in Table 1. The reference strain *E*. *coli* W3110 is an F^-^ λ^-^ galactose fermenting derivative of *E. coli* K-12 [48]. Strain VH32 is a Δ*ptsI, ptsH, crr*::*kan* Δ*lacI, lacZ*::*loxP* (PTS^-^) derivative of W3110 displaying a PTS^-^ glc^-^ phenotype [49]. Strain VH32 *galP*^+^ is a derivative of VH32 that was constructed by replacing the native galactose permease gene (*galP*) promoter by the *trc* promoter [50]. The method employed for this purpose leaves a stable chloramphenicol (Cm) resistance cassette (*cat*) flanked by phage λ Red recombinase (FLP) recognition target (FRT) sites [51]. In this strain, glucose import is dependent on GalP and glucose phosphorylation on glucokinase (Glk) activity encoded by the chromosomal *glk* gene. Since the *lacI* gene was deleted in strain VH32 *galP*^+^, expression of *galP* from the *trc* promoter is constitutive. To generate strain VH33, the *cat* resistance cassette was removed from VH32 *galP*^+^ by transforming it with plasmid pCP20 that expresses FLP [52]. Strains VH34 (Δ*ptsI, ptsH, crr*::*kan*, *pykA*::*cat*) and VH35 (Δ*ptsI, ptsH, crr*::*kan*, *pykF*::*aacC1*) were generated by P1 transduction of VH33 using PB28 strain (Δ*ptsI, ptsH, crr*::*kan*, *pykA*::*cat*, *pykF*::*aacC1*) [28] lysate as a donor and selecting with the corresponding antibiotic: chloramphenicol (Cm) or gentamicin (Gen). Verification of the desired genotype was made by PCR; in the case of *pykA* interruption by *cat* the 5’ primer was TGA AGG AAT CGC GTT TTG ATA and the 3’ primer: GTA TTA GTA GAA CCC ACG GTA. For verification of *pykF* interruption with *aacC1* the PCR reaction included the 5’ primer: CGT AAC CTT TTC CCT GGA ACG and the 3’ primer: GAG TCC GGC TGA TGT TGG GAG TAG GTG GCT. *pck*^-^ derivatives were generated by P1 transduction in W3110, VH33, and VH35 using a JM101 *pck*^-^ strain [53] (s*up*E, *thi*, Δ(*lacpro*AB), F’, *pck*::*cat*) lysate as donor and selecting with Cm. In these *pck*^-^ strains the *cat* cassette is flanked by FRT sites, so in order to generate strain VH34 *pck*^-^, the *cat* resistance cassette was removed from VH33 *pck*^-^ strain by transforming it with plasmid pCP20, and then performing P1 transduction in the resulting strain using a lysate from PB28 (Δ*ptsI, ptsH, crr*::*kan*, *pykA*::*cat*, *pykF*::*gen*) as a donor. Resulting strain VH33 *pck*^-^*pyk*A^-^ (VH34 *pck*^*-*^) strain was selected using medium with Cm. Verification of the *pck::cat* mutation in derivative strains was done by PCR using primers reported elsewere [54] (data not shown). Two plasmids were used in this work (Table 1). pJLB*aroG*^fbr^*tktA*[55] is a pACYC184 derivative that harbors gene *aroG*^fbr^ encoding a feedback resistance version of DAHP synthase. This plasmid also contains a copy of the gene *tkt*A that encodes transketolase, an enzyme that catalyzes the reversible conversion of D-fructose-6-phosphate (F6P) and D-glyceraldehyde-5-phosphate (G5P) into D-xylulose-5-phosphate (XU5P) and E4P. The *aro*G^fbr^ gene is under transcriptional control of the *lac*UV5 strong promoter that is inducible by iso-propyl-β-D-thiogalactopyranoside (IPTG), whereas the *tkt*A gene is under control of its native promoter. Plasmid p*TrcpheA*^ev2^ contains a second evolved version of a mutant version of chorismate mutase-prephenate dehydratase enzyme (PheA) [32]. This gene is under transcriptional control of the the *trc* promoter. Strains W3110, VH33, VH34 and VH35 were sequentially transformed with plasmids pJLB*aro*G^fbr^*tktA* and p*TrcpheA*^ev2^ to generate strains W3110A, VH33A, VH34A and VH35A, respectively. 

**Table 1:** ***Escherichia coli*strains and plasmids used**

**Strain**	**Description**	**Reference**
PB28	Δ*ptsI, ptsH, crr*::*kan, pykA*::*cat, pykF*::*gen*	[28]
JM101 *pck*^-^	*Sup*E, *thi*, Δ(*lac*-*pro*AB), F’, *pck*::*cat*	[53]
W3110	F^-^ λ^-^ INV(*rrn*D - *rrn*E)1	[48]
VH32 *galP*^*+*^	W3110 ΔptsH, ptsI, crr::Km, ΔlacI, lacZ::loxP ΔP_*galP*_::*cat*,P_*Trc*_	[50]
VH33	VH32 *galP*^*+*^ ΔP_*galP*_:: P_*Trc*_	This work
VH34	VH33 *pykA*::*cat*	This work
VH35	VH33 *pykF*::*gen*	This work
W3110 *pck*^-^	W3110 *pck*::*cat*	This work
VH33 *pck*^-^	VH33 *pck*::*cat*	This work
VH34 *pck*^-^	VH34 *pck*::*cat*	This work
VH35 *pck*^-^	VH35 *pck*::*cat*	This work
W3110A	W3110/JLB*aroG*^fbr^*tktA, pTrcpheA*^*ev2*^	This work
VH33A	VH33/JLB*aroG*^fbr^*tktA, pTrcpheA*^*ev2*^	This work
VH34A	VH34/JLB*aroG*^fbr^*tktA, pTrcpheA*^*ev2*^	This work
VH35A	VH35/JLB*aroG*^fbr^*tktA, pTrcpheA*^*ev2*^	This work
Plasmids		
pCP20	FLP^+^, λci857^+^, λ p_R_Rep^ts^, Ap^R^, Cm^R^	[52]
pJLB*aroG*^fbr^*tktA*	*aroG*^fbr^ under control of the *lacUV5* promoter; and *tktA* under its native promoter, carries *lacIq* and *tet* genes. Replication origin from pACYC184	[55]
p*TrcpheA*^ev2^	Evolved *pheA*^ev2^ under the control of *lacUV5* promoter. Ev2 superscript means 2nd version of evolved *pheA*^fbr^ gene.	[32]

### Growth media and cultivation conditions

The medium used during mutant strains construction and selection was Luria-Bertani (LB) with the corresponding antibiotic: Cm 10 μg/ml, Km 10 μg/ml and Gm 5 μg/ml (Table 1). In shake flask cultures and labeling experiment, minimal medium was used. It contained glucose as the sole carbon source at a concentration of 10 g/l, K_2_HPO_4_ 90 mM, KH_2_PO_4_ 10 mM, (NH_4_)_2_SO_4_ 40 mM, NaCl 20 mM, MgSO_4_·2H_2_O 1.6 mM, CaCl_2_ 0.05 mM, FeSO_4_·7H_2_O 0.072 mM and vitamin B1 0.05 mM. Glucose and salts solution were sterilized separately at 121°C during 20 minutes and vitamin B1 was sterilized by filtration. All stock cultures were stored at −70°C in LB medium containing glycerol (25% v/v). For fermentation profiles, 5 ml of LB overnight cultures were used as inoculum, washed twice with minimal medium and then sub-cultured in shake flask containing 30 ml of minimal medium with 5 g/l glucose starting with an OD_600nm_ = 0.1 and incubated at 37°C, 300 rpm during 12 hours in an orbital shaker (G25, New Brunswick Scientific, Inc., New Brunswick, NJ). These cultures were used to inoculate 30 ml of minimal medium with 10 g/l glucose at an OD_600nm_ = 0.01 and then incubated at 37°C, 300 rpm during 24 hours. Every 2 hours 1 ml of culture was harvested, OD was determined (Biomate 5, ThermoSpectronic), cells were centrifuged (10,000 g for 10 min) and the supernatant used for metabolite determination by HPLC. For labeling experiments, the naturally labeled glucose was substituted by 99% [1- ^13^C] labeled glucose (Cambridge Isotope Laboratories, Andover, USA). Cells were harvested during the exponential growth phase at 9 hours (OD_600nm_ = 3.72) for W3110, 15 hours (OD_600nm_ = 6.68) for VH33, 16 hours for VH34 (OD_600nm_ = 7.68) and 16 hours for VH35 (OD_600nm_ = 5.1). Cultures for determining the correlation between optical density (600 nm) and cell dry mass (CDM) were started with 5 ml of LB overnight cultures that were used as inoculum, washed twice with minimal medium and then used to inoculate a shake flask containing 50 ml of minimal medium with glucose 5 g/l starting at an OD_600nm_ = 0.1 and incubated at 37°C, 300 rpm for 12 hours in an orbital shaker. To determine the correlation between optical density and CDM, 10 ml of cells grown in minimal medium were harvested and filtered at different optical densities, washed twice with water and dried at 80°C until constant weight. The correlation between OD_600nm_ and CDM [g/L] was determined as CDM = 0.45 x OD_600nm_.

### Sample preparation for labeling analysis and estimation of metabolic flux

The total volume of cultures grown with [1- ^13^C] labeled glucose (10 ml) was centrifuged and the pellet (about 1 mg dry cell mass) was washed twice with cold water (4°C). Hydrolysis was performed with 50 μL of HCl 6 N at 110°C for 24 hours. The insoluble debris was separated by filtration (Titan 2 HPLC filter purple, 0.2 μm nylon membrane, Sun-Sri), neutralized with NaOH 6 N and lyophilized. Derivatization with (trimethylsilyl) trifluoroacetamide (BSTFA) of the lyophilized cell protein was performed to determine ^13^C labeling pattern of proteinogenic amino acids by GC-MS as previously described [56]. The metabolic network for simulation was comprised of the EMP pathway, the PPP, the TCA cycle and the Ender Duodoroff pathway. Glyoxylate shunt, anaplerotic reactions and malic enzyme were included. The simulation was done as previously described [56].

### Resting cells experiments

To determine PHE production and yield in W3110A, VH33A, VH34A and VH35A strains, a resting cells method was used. 5 ml of LB overnight cultures were used as inoculum, washed twice with minimal medium supplemented with 10 g/l of yeast extract (MM + YE) and then sub-cultured in shake flask containing 50 ml of MM + YE with 5 g/l glucose starting with an OD_600nm_ = 0.1 and incubated at 37°C, 300 rpm until optic density of 2.0. At this point, cells were harvested, washed twice with minimal medium and suspended in 50 ml of minimal medium with 10 g/l glucose. To arrest cellular growth 50 μg/ml of chloramphenicol was added as well as 100 μM IPTG for *aro*G^fbr^ and *phe*A^ev2^ transcriptional induction. Cell cultures were incubated during 12 hours in an orbital shaker. Every hour, 1 ml of culture was harvested, OD_600nm_ was determined, cells were centrifuged (10,000 g for 10 min) and the supernatant used for metabolite determination by HPLC.

### Determination of glucose, organic acids, DHS, SHIK and PHE

For determination concentrations of D-glucose and organic acids, an Aminex HPX-87 H column (300 X 7.8 mm; 9 Am Bio-Rad, Hercules, CA) was used. Separation was carried out isocratically with 5 mM H_2_SO_4_ at a flow rate of 0.5 ml/min and a temperature of 50°C. Under these conditions glucose was detected by refraction index and acetic acid, DHS and SHIK by photodiode array at 210 nm. For these measurements a Waters HPLC system was used: 600E quaternary pump, 717 automatic injector, 2410 refraction index, and 996 photodiode array.

PHE concentration in culture supernatants was quantified with a Phenomenex Synergy Hydro RP18 column (150 by 4.6 mm; 4 m) attached to an Agilent 1100 HPLC system (Agilent Technologies, Palo Alto, CA). Running conditions were: mobile phase, 0.2% TFA in 40% methanol; flow, 0.5 ml/min. Detection was performed with a photodiode array at 280 nm.

### Determination of Pyk enzyme activity

Cells in exponential growth phase were harvested by centrifugation (10,000 g for 10 min), washed twice with 50 mM sodium phosphate buffer (pH 7.2 at 4°C), suspended in the same buffer, and disrupted by sonication using four 15-second, λ = 10 μm, pulses in a cold bath (Ultrasonic Disrupter, Soniprep 150). The cell debris was removed by centrifugation (10,000 g, 10 min, 4°C), and the resulting crude cell extracts were immediately used to determine enzyme activities. Pyk enzyme activities were determined spectrophotometrically (Biomate 5, ThermoSpectronic) using 1 U of lactate dehydrogenase (LDH) as coupling enzyme [57]. The reaction mix contained KCl 50 mM, MgCl_2_ 5 mM, ADP 2 mM, NADH 0.2 mM, PEP 100 mM, fructose-1,6 biphosphate (FBP) 1 mM, AMP 1 mM and ribose-5-phosphate (R5P) 1 mM. One unit of specific enzyme activity was defined as the amount of enzyme required to convert 1 μmol of substrate into the specific product per minute per milligram of protein at 30°C. Protein concentrations were measured using the Lowry method. Enzyme activities reported are the average of three independent experiments.

## Results

### Pyk mutant strains verification

Strain VH33 is a W3110 mutant derivative lacking PTS activity as a result of deleting genes *ptsI*, *ptsH* and *crr*. This strain was also modified in order to increase its glucose import capacity by replacing the native promoter of gene *galP* by the strong *trc* promoter and inactivating the *lacI* gene. As a result of these modifications VH33 displays a constitutive PTS^-^ glc^+^ phenotype. In this work, strain VH33 was further modified by inactivating either *pykA* or *pykF*, to generate strains VH34 and VH35, respectively.

**Figure 3:**
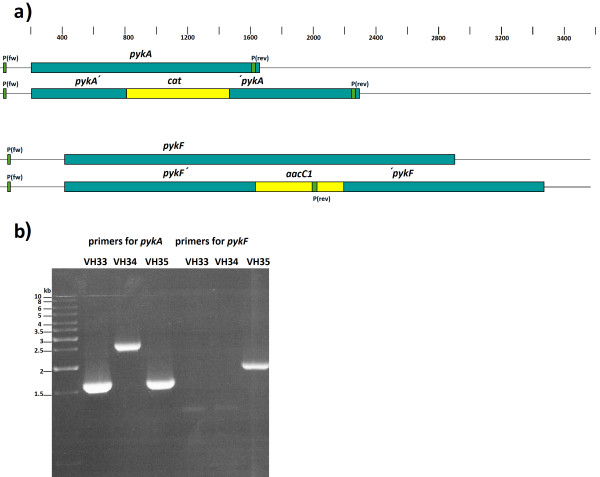
**Mutant strain verification.** (**a**) Chromosomal regions of genes *pyk*A, *pyk*F, *pyk*A::*cat* and *pyk*F::*aac*C1. The annealing sites of the primers employed for strain verification are depicted (green color). (**b**) Gel electrophoresis of the genomic DNA PCR products of strains VH33 (PTS^-^), VH34 (PTS^-^*pyk*A^-^) and VH35 (PTS^-^*pyk*A^-^).

Figure 3a depicts the genomic regions of *pykA* and *pykF* and the resulting modifications after interruption with *cat* (VH34) or *aacC1* (VH35) antibiotic resistance genes. The forward primer for verification of *pykA* interruption anneals outside of the *pykA* region while the reverse primer binds at the end of the gene. PCR amplification using genomic DNA from VH33, VH34 and VH35 as template showed two expected bands: one of 1.6 kb for VH33 and VH35 and one of 2.3 kb for VH34 due to the insertion of the *cat* gene in the *pykA* gene (Figure 3b). For verifying strain VH35, the forward primer employed anneals outside of the *pykF* coding region while the reverse primer binds to the *aacC1* gene. When using genomic DNA from VH33, VH34 and VH35 as template, no PCR product was observed for VH33 or VH34 while the expected 2 kb band was observed for VH35 (Figure 3b). These results, together with the expected antibiotic resistance phenoptypes, indicated that interruptions of *pykA* by *cat* and *pykF* by *aacC1* were present in VH34 and VH35.

### Measurement of Pyk enzyme activity

**Figure 4:**
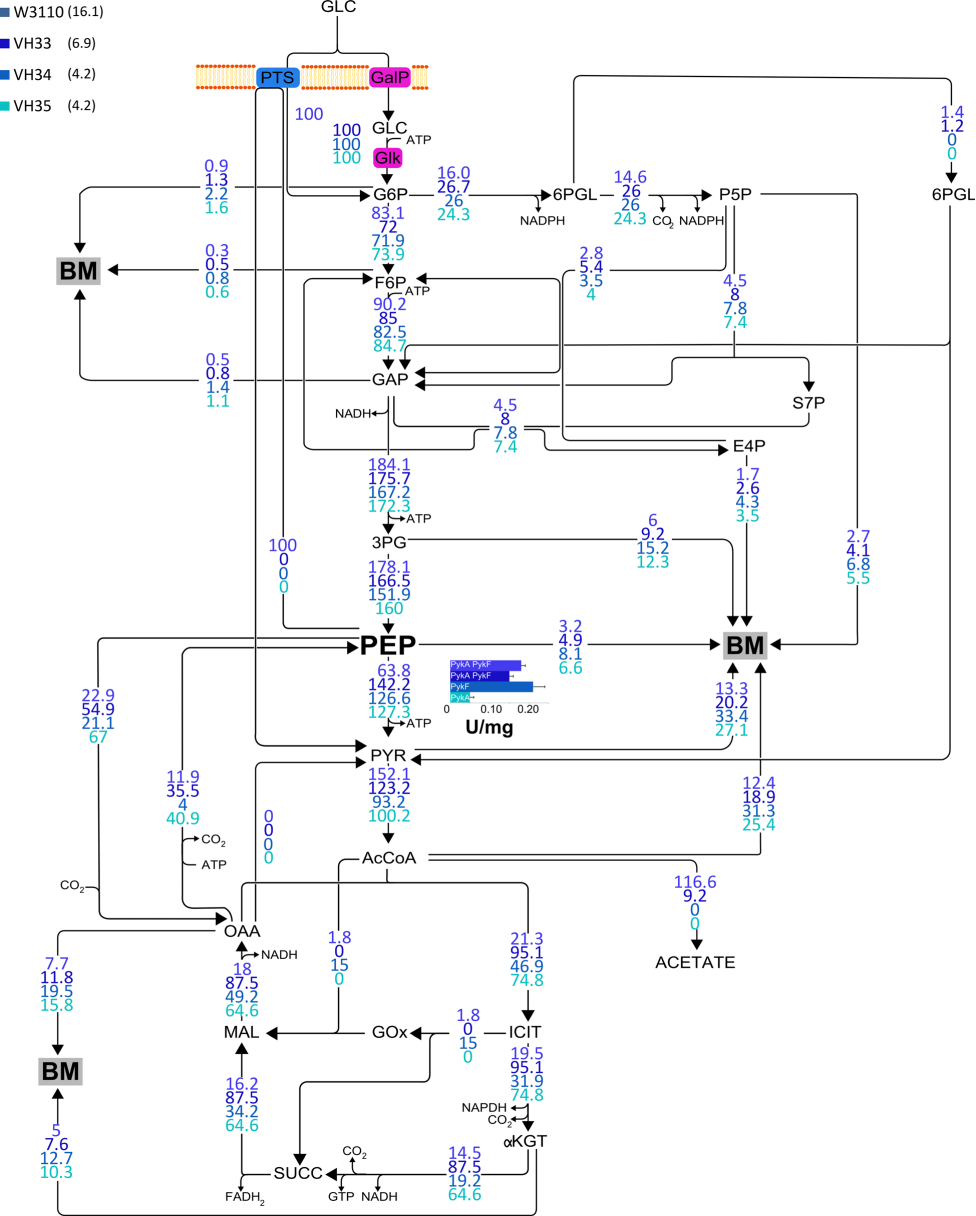
**Metabolic flux distribution and Pyk activity.** Metabolic flux distribution and Pyk activities of strains W3110 (reference), VH33 (PTS^-^ glc^+^), VH34 (PTS^-^ glc^+^*pykA*^-^) and VH35 (PTS^-^ glc^+^*pykF*^-^) (from dark to light blue respectively). The figure shows the relative flux of each strain. Between PEP and PYR nodes a graph bar of Pyk activity is depicted. In parenthesis, next to the strains names, the specific glucose consumption rates (mmol / g · h) are shown.

In the wild type strain W3110 the activities that determine the PEP → PYR flux are PTS, PykA and PykF, while in strain VH33 this reaction is catalyzed only by both Pyk isozymes. In strains VH34 and VH35, the PEP → PYR reaction is dependent only on PykF or PykA, respectively. The measured total Pyk enzyme activity (PykA + PykF) in strain W3110 was 0.18 ± 0.03 U/mg and a similar value was observed in strain VH33 (Figure 4). In strain VH34, where only the PykF isozyme is active, the Pyk specific activity was 0.21 ± 0.01 U/mg, whereas in the VH35 strain the Pyk activity, dependent only on PykA, was 0.05 ± 0.01 U/mg (Figure 4).

### Batch culture profiles

To determine the effects on kinetic and stoichiometric parameters caused by PTS, PTS *pykA* and PTS *pykF* inactivations, batch cultures in minimal medium with 10 g/l of glucose as sole carbon source were performed (Table 2). Under these conditions, reference strain W3110 displayed a μ of 0.69 h^-1^ and a q_Glc_ of 16.1 mmol/g/h; both values were the highest among the tested strains. A high q_Ac_ of 4.31 mmol/g/h was determined and no other by-products were observed. The biomass yield from glucose (Y_x/s_) was 52.44 g/ mol, the lowest value observed among strains in this study. Strain VH33 displayed a μ of 0.45 h^-1^ and a specific glucose uptake rate (q_Glc_) of 6.9 mmol/g/h. The specific acetate production rate (q_Ac_) was 5% of the value observed in W3110. When compared to the wild type strain, the Y_x/s_ in VH33 increased 20%. Deletion of *pykA* (VH34) had no significant effect on the μ (0.44 h^-1^), whereas deletion of *pykF* (VH35) had a negative effect (0.36 h^-1^). Both strains showed the same q_Glc_ value (4.2 mmol/g/h) and no acetate production was detected. The highest Y_x/s_ value among all tested strains was observed for strain VH34 (Table 2).

**Table 2:** Growth kinetic parameters in minimal media

**Strain**	**μ (h**^**-1**^**)**	**Y**_**x/s**_**(g / mol)**	**q**_**s**_**(mmol / g / h)**	**Y**_**Ac/Glc**_**(mol / mol)**	**q**_**Ac**_**(mmol / g/ h)**
**W3110 (WT)**	0.69 ± 0.01	52.44 ± 4.42	16.1 ± 0.33	1.2 ± 0.01	4.31 ± 0.12
**VH33 (PTS**^**-**^**)**	0.45 ± 0.00	63 ± 3.60	6.9 ± 0.61	0.09 ± 0.00	0.23 ± 0.00
**VH34 (PTS**^**-**^**pykA**^**-**^**)**	0.44 ± 0.00	104.4 ± 16.20	4.2 ± 0.61	--	--
**VH35 (PTS**^**-**^**pykF**^**-**^**)**	0.36 ± 0.01	84.6 ± 9.00	4.2 ± 0.41	--	--
**W3110*pck***^**-**^	0.64 ± 0.04	51.76 ± 1.93	12.44 ± 0.23	0.32 ± 0.03	1.14 ± 0.01
**VH33*pck***^**-**^**(PTS**^**-**^**pck**^**-**^**)**	0.37 ± 0.00	80.47 ± 0.86	4.61 ± 0.09	0.01 ± 0.01	0.03 ± 0.00
**VH34*pck***^**-**^**(PTS**^**-**^**pykA**^**-**^**pck**^**-**^**)**	0.33 ± 0.02	101.00 ± 2.87	3.35 ± 0.13	--	--
**VH35*pck***^**-**^**(PTS**^**-**^**pykF**^**-**^**pck**^**-**^**)**	0.29 ± 0.00	73.32 ± 2.06	3.96 ± 0.13	--	--

### Metabolic flux responses to PTS inactivation

To determine in fine detail the consequences of PTS and PykA or PykF inactivation on central carbon metabolism, ^13^C metabolic flux analysis was performed. In reference strain W3110, the EMP pathway was identified as the main metabolic route involved in glucose catabolism, the PPP had a minor role and the Entner-Duodoroff pathway (EDP) was hardly active. The TCA cycle displayed a low carbon flux value and the glyoxylate shunt was slightly active (Figure 4). It is noteworthy that a low level of carbon exchange was detected between PEP and OAA (catalyzed by Ppc and Pck), forming a cycle where one ATP molecule is hydrolyzed.

PTS inactivation had a strong effect on the metabolic flux distribution throughout the whole central metabolic network. The EMP pathway relative flux was reduced while PPP and TCA cycle fluxes were increased. When compared to data from strain W3110, an increase in the exchange rate between PEP and OAA, involving Ppc and Pck, was observed. Also, strain VH33 displayed higher carbon flux to biomass when compared to the wild-type strain. At the lower part of the EMP pathway, the total PEP → PYR flux decreased from 163% in W3110 to 142% in VH33; however, the flux dependent only on Pyk isozymes activity increased from 63.8% in W3110 to 142.2% in the PTS^-^ glc^+^ strain. The flux from acetyl coenzyme A (AcCoA) to acetate was reduced from 116% in W3110 to 9% in VH33. When compared to W3110, a 4 fold higher flux into the TCA was observed in strain VH33.

### Metabolic flux responses to Pyk isozyme inactivation in the PTS^-^ glc^+^ background

Inactivation of either Pyk isozyme in the PTS^-^ glc^+^ background caused a similar level of reduction in q_s_ and q_ac_, and the magnitude of the flux from PEP to PYR was similar in both strains. However, values for μ, Yx/s and Pyk activity, differed between strains VH34 and VH35. Compared to strain VH34, the anaplerotic flux from PEP to OAA was 3 fold higher in VH35, and the flux directed to the TCA cycle via GltA was also higher (Figure 4). Flux from AcCoA to acetate was absent in both strains and the glyoxylate shunt was found to be active only in VH34. A high carbon exchange flux between PEP and OAA was found in VH35, whereas for VH34 the exchange flux value was the lowest of all studied strains. When compared to strain VH33, it was observed that inactivation of either Pyk isozyme caused an increase in carbon flux directed to biomass formation.

### Physiological responses to Pck inactivation

The flux analysis data revealed the existence of a metabolic cycle involving Ppc and Pck in all studied strains. In order to gain more insight into the role of this cycle, we generated *pck*^-^ mutant derivatives of W3110, VH33, VH34 and VH35. When these derivative strains were cultured in minimal medium with glucose as sole carbon source, a reduction in μ and qs was observed. Also, a 75% and 87% reduction in the rate of acetate production was observed in strains W3110 and VH33, respectively (Table 2).

### Aromatic amino acids production

The effects of PTS and Pyk inactivation on aromatics production capacity, was determined on strains W3110A, VH33A, VH34A and VH35A that carry plasmids pJLB*aroG*^fbr^*tkt*A and p*TrcpheA*^ev2^ having genes encoding enzymes that increase carbon flow from central metabolism to the common aromatic and PHE biosynthetic pathways. These modifications cause an increase in PHE production capacity. However, accumulation and secretion of pathway intermediates such as DHS and SHIK also occurs as consequence of pathway bottlenecks in the common aromatic pathway.

Metabolites DHS and SHIK as well as PHE, were quantified from supernatants of resting cells cultures. Figure 2a and Table 3 show the calculated yields from glucose for DHS, SHIK and PHE, as well as the sum of these three compounds (Y_DHS+SHIK+PHE/Glc_). A yield value that includes the sum of the amounts detected for DHS, SHIK and PHE is the best indicator of aromatics production capacity, which itself is directly related to PEP biosynthetic availability. Inactivation of PTS caused a near 10-fold increase in Y_DHS+SHIK+PHE/Glc_ when compared to wild-type strain W3110. This is the result of replacing PEP-dependent glucose transport in the wild-type strain by an ATP-dependent mechanism in strain VH33 [23,30]. In the PTS^-^ glc^+^ background, inactivation of PykA (VH34) or PykF (VH35) caused 3.4 and 6.6-fold increases in Y_DHS+SHIK+PHE/Glc_ when compared to W3110. 

**Table 3:** Batch profiles and common shikimate pathway intermediaries and phenylalanine yields from glucose of W3110A, VH33A, VH34A and VH35A resting cells in minimal media

**Strain**	**q**_**s**_**(mmol / g / h)**	**q**_**Ac**_**(mmol / g/ h)**	**Y**_**DHS / Glc**_**(mol / mol)**	**Y**_**SHIK / Glc**_**(mol / mol)**	**Y**_**PHE / Glc**_**(mol / mol)**	**Y**_**DHS+SHIK+PHE / Glc**_**(mol / mol)**
**W3110A**	2.51 ± 0.10	7.52 ± 0.46	0.01 ± 0.00	0.00 ± 0.00	0.04 ± 0.01	0.05 ± 0.01
**VH33A**	0.70 ± 0.04	--	0.13 ± 0.03	0.06 ± 0.01	0.25 ± 0.03	0.44 ± 0.06
**VH34A**	1.96 ± 0.02	--	0.05 ± 0.01	0.04 ± 0.00	0.08 ± 0.00	0.17 ± 0.01
**VH35A**	0.91 ± 0.04	--	0.24 ± 0.01	0.06 ± 0.00	0.03 ± 0.01	0.33 ± 0.02

## Discussion

The objective of the present work consisted on determining the effects of PTS and Pyk isozymes inactivation on cell physiology, metabolic flux distribution and PEP availability for aromatics biosynthesis. The inactivation of PTS in *E. coli* abolishes PEP-dependent glucose transport; therefore PYR production from PEP is dependent only on Pyk isozyme activities. In this study, by inactivating each Pyk isozyme in a PTS^-^ glc^+^ background, strains were generated where the PEP to PYR reaction was dependent only on PykA or PykF activity. These strains were characterized by flux analysis, thus providing the first quantitative description of the metabolic consequences of the sequential elimination of activities catalyzing the PEP to PYR reaction.

The inactivation of PTS in *E. coli* causes a strong reduction in q_Glc_ and μ, therefore, such mutant strains display a PTS^-^ glc^-^ phenotype. To improve q_Glc_ and μ, strain VH33 has a chromosomal modification that increases its capacity for non PTS-dependent glucose transport and thus displays a PTS^-^ glc^+^ phenotype. In strain VH33 glucose phosphorylation is not dependent on PEP; instead, ATP is the phosphate donor in a reaction catalyzed by glucokinase. The μ of the PTS^-^ glc^-^ progenitor strain VH32 is 0.03 h^-1^, whereas this value increased to 0.45 h^-1^ in VH33 as a result of replacing the native promoter of gene *galP* with the strong promoter *trc*[50]. In this study, the q_Glc_ of strain VH33 was determined and it corresponded to 43% of that measured in wild-type W3110. An expected consequence of the lower q_Glc_ in VH33 is a decreased flux in the EMP pathway. The flux analyses performed in this report revealed that the main observed response to PTS inactivation was flux rerouting at the AcCoA node, resulting in a highly reduced q_Ac_. This metabolic response is expected to compensate for the reduced glycolytic flux in strain VH33, diverting the AcCoA not consumed for acetate production mainly into the TCA cycle. The net result is an augmented flux into the TCA cycle with an expected increase in the synthesis rate of biomass precursors αKGT and OAA, as well as reduced cofactors that can lead to ATP synthesis by oxidative phosphorylation. Inactivation of either PykA or PykF in the PTS^-^ glc^+^ background caused an approximately further 50% reduction in q_Glc_ and flux in the PEP to PYR reaction when compared to data from VH33. These results indicate that both isozymes should be active to sustain the high flux level in this part of the EMP pathway when *E. coli* grows on glucose. Acetate production was not detected in strains VH34 and VH35. In these strains, the relative flux to the TCA cycle was higher than that observed for W3110 but lower than the value for VH33. A second observed response to PTS or PTS and Pyk inactivation consisted in flux rerouting in the central metabolic network leading to increased flux to biomass generation. The flux value to biomass was nearly twofold higher in strains VH34 and VH35, when compared to VH33. This metabolic response can be expected to have the effect of conserving the reduced amount of intermediate metabolites resulting from the diminished glucose import and catabolism capacity in the PTS^-^ and Pyk^-^ mutant strains. As expected, the flux values leading from the central metabolic pathways to biomass formation showed a positive correlation with the observed Y_x/s_ in all studied strains.

Evidence of cycling via Ppc and Pck was detected in the wild-type strain W3110. This metabolic cycle has also been reported by other authors in wild-type *E. coli*[26,27]. A comparison among the mutant strains generated in this study provides the opportunity to explore the role of this cycle in *E. coli*. Flux analysis revealed that PTS inactivation causes an increase in the cycle net flux. A similar response is observed by simultaneous PTS and PykF inactivation (strain VH35). In contrast, the lowest net flux value was observed in strain VH34 that lacks PTS and PykA activities. These results suggest a partial inverse correlation between q_s_ and the net flux in this metabolic cycle, which can be observed when comparing strains W3110, VH33 and VH35. It is not clear why this correlation does not apply for strain VH34. This might be related to the differential effects of PykA or PykF inactivation on the metabolic flux distribution around the PEP node. In a previous report, when comparing a wild-type strain and a derivative lacking both PykA and PykF activities, the Pyk double mutant displayed a higher cycle net flux [26]. However, in this case, the q_Glc_ of the strains were similar, so the observed effect must be mainly related to the reduced flux in the PEP to PYR reaction.

The ATP/ADP ratio is an important physiologic parameter that modulates several cellular functions. It has been observed that a reduction in the ATP/ADP ratio, caused by increasing ATP hydrolysis activities, stimulates flux in the EMP pathway [58]. Each cycle involving Ppc and Pck activities consumes one ATP molecule. Thus, a reduction in the cellular ATP/ADP ratio could be expected at high Ppc-Pck cycle flux values, and this could stimulate EMP pathway flux. The observed inverse correlation between q_Glc_ and the net cycle flux in strains W3110, VH33 and VH35 suggests this cycle might be contributing to increasing EMP pathway flux and thus, growth capacity in the mutant strains. To test this hypothesis, mutant derivatives of strains W3110, VH33, VH34 and VH35 lacking Pck activity were constructed and characterized. A reduction in q_Glc_ and μ was observed in all derivative Pck^-^ strains, suggesting that the Ppc-Pck cycle's function is related to stimulating flux in the EMP pathway.

Some of the metabolic responses discussed above, that include q_Glc_ reduction and flux rerouting at several points in central metabolism can be partially explained considering that PEP has a role in the allosteric regulation of several enzymes of the EMP pathway. In addition, inhibition of glucokinase by PEP has been reported [9]. Considering the genetic modifications performed, a larger PEP pool would be expected in strains VH33, VH34 and VH35 when compared to W3110. This assumption is consistent with the higher metabolic PEP availability demonstrated in PTS^-^ glc^+^ strains that have been shown to produce aromatic compounds with a higher yield from glucose and with data from PTS- Pyk- mutats that will be discussed below [23,30].

Strains W3110A, VH33A, VH34A and VH35A carry plasmids with genes encoding enzymes that increase carbon flow into the common aromatic and the PHE biosynthetic pathways (Figure 2a and Table 3). When considering the Y_DHS+SHIK+PHE/Glc_ from resting cells experiments in minimal medium with glucose, it can be observed, as expected, that PTS inactivation causes an increase in this parameter. In the PTS^-^ glc^+^ background, inactivation of PykA (VH34A) or PykF (VH35A), would be expected to further increase PEP availability for aromatics biosynthesis. However, the Y_DHS+SHIK+PHE/Glc_ value was lower in these two strains when compared to VH33A. These results can be explained considering that in strains VH34 and VH35 a higher flux value is directed from central metabolism to biomass formation when compared to VH33. In strains VH34 and VH35, an inverse correlation was observed between Y_x/Glc_ and Y_DHS+SHIK+PHE/Glc_. These data indicates that a carbon-limitation state in minimal medium with glucose reduces PEP availability for aromatics production in strains VH34 and VH35 as a result of competition for central metabolic precursors, including PEP, between biomass formation reactions and the modified aromatic pathways in stains VH34A and VH35A. Consumption of precursors for biomass synthesis from central metabolism could be reduced in these two strains if they are provided in the culture medium. When resting cells experiments were performed in minimal medium supplemented with yeast extract (10 g/l), a 19, 14 and 25-fold increase was observed in VH33A, VH34A and VH35A in Y_DHS+SHIK+PHE/Glc_ values when compared with W3110 (data not shown). In addition, two reports have shown that strains with PTS^-^ glc^+^ PykA^-^ or PTS^-^ glc^+^ PykF^-^ phenotypes display higher production capacity for aromatic compounds in complex media when compared to an isogenic PTS^-^ glc^+^strain [17,22].

It has been shown that a strain having the same phenotype as VH33 displays a better production performance for recombinant protein production when compared to W3110 [50]. This result can be explained by the lower q_Ac_ and higher Y_x/Glc_ displayed by the PTS^-^ glc^+^ mutant, when compared to the wild-type strain. In this regard, it is noteworthy that inactivation of either Pyk isozyme in VH33 caused a further reduction in Y_Ac/Glc_ to the point where this organic acid was not detected by HPLC. Interestingly, strain VH34 displayed no acetate production, a 66% increase in Y_x/s_ over VH33 and the same μ. In a recent report, strain VH34 transformed with a plasmid DNA vaccine (pDNA) and grown in complex medium supplemented with glucose produced 50% less acetate and nearly 4-fold more pDNA when compared to strain VH33/pDNA [59]. These data shows that strain VH34 has advantageous characteristics for a microbial host that could be employed for the development of fermentative process for plasmid DNA or recombinant protein production.

## Conclusions

In this study, the contribution of PTS and each of the Pyk isozymes to the PEP to PYR flux as well as the consequences of their inactivation on central metabolism were determined by metabolic flux analysis. This work showed that the presence of either one of the two Pyk isozymes generates quite different physiological and central carbon metabolism flux responses. The general observed response to PTS or Pyk inactivation is consistent with central metabolism flux rerouting to compensate for the lower EMP flux observed in these mutant strains. The detailed characterization presented here provides further insight into the flexibility of the central metabolic network in *E. coli* and will be useful in the design of strategies for generating strains for the production of aromatic metabolites, plasmid DNA or recombinant proteins.

## Abbreviations

### Metabolic pathways

EMP: Embden Meyerhof Parnas; TCA: Tricarboxylic acid cycle; PPP: Pentose pathway; GalP: Galactose permease; Gap: Glyceraldehydes 3-phosphate dehydrogenase; Glk: Glucokinase; GltA: Citrate synthase; Gnd: 6-phosphogluconate dehydrogenase; Mdh: Malate dehydrogenase; Mez: NAD- and NADP dependent malic enzymes; Pck: Phosphoenolpyruvate carboxykinase; Pgi: Phosphoglucose isomerase; Ppc: Phosphoenolpyruvate carboxylase; PTS: Phosphoenolpyruvate:carbohydrate phosphotransferase system; PykAF: Pyruvate kinases A and F; Zwf: Glucose 6-phosphate dehydrogenase; AcCoA: Acetyl-coenzyme A; CIT: Citrate; E4P: Erythose 4-phosphate; F6P: Fructose 6-phosphate; αKGT: α-ketoglutarate; GAP: Glyceraldehydes 3-phosphate; G6P: Glucose 6-phosphate; Glc: Glucose; GOx: Glyoxylate shunt; ICIT: Isocitrate; MAL: Malate; NADPH: Nicotinamide adenine dinucleotide phosphate (reduced form); NADH: Nicotinamide adenine dinucleotide (reduced form); OAA: Oxaloacetate; 2PG: 2-phosphoglycerate; 3PG: Phosphoglycerate; 6PGL: 6-phosphogluconolactone; PEP: Phosphoenolpyruvate; PYR: Pyruvate; R5P: Ribose 5-phosphate; SUCC: Succinate; BM: Biomass.

### Enzymes

EMP: Embden Meyerhof Parnas; TCA: Tricarboxylic acid cycle; PPP: Pentose pathway; GalP: Galactose permease; Gap: Glyceraldehydes 3-phosphate dehydrogenase; Glk: Glucokinase; GltA: Citrate synthase; Gnd: 6-phosphogluconate dehydrogenase; Mdh: Malate dehydrogenase; Mez: NAD- and NADP dependent malic enzymes; Pck: Phosphoenolpyruvate carboxykinase; Pgi: Phosphoglucose isomerase; Ppc: Phosphoenolpyruvate carboxylase; PTS: Phosphoenolpyruvate:carbohydrate phosphotransferase system; PykAF: Pyruvate kinases A and F; Zwf: Glucose 6-phosphate dehydrogenase; AcCoA: Acetyl-coenzyme A; CIT: Citrate; E4P: Erythose 4-phosphate; F6P: Fructose 6-phosphate; αKGT: α-ketoglutarate; GAP: Glyceraldehydes 3-phosphate; G6P: Glucose 6-phosphate; Glc: Glucose; GOx: Glyoxylate shunt; ICIT: Isocitrate; MAL: Malate; NADPH: Nicotinamide adenine dinucleotide phosphate (reduced form); NADH: Nicotinamide adenine dinucleotide (reduced form); OAA: Oxaloacetate; 2PG: 2-phosphoglycerate; 3PG: Phosphoglycerate; 6PGL: 6-phosphogluconolactone; PEP: Phosphoenolpyruvate; PYR: Pyruvate; R5P: Ribose 5-phosphate; SUCC: Succinate; BM: Biomass.

### Compounds and others

EMP: Embden Meyerhof Parnas; TCA: Tricarboxylic acid cycle; PPP: Pentose pathway; GalP: Galactose permease; Gap: Glyceraldehydes 3-phosphate dehydrogenase; Glk: Glucokinase; GltA: Citrate synthase; Gnd: 6-phosphogluconate dehydrogenase; Mdh: Malate dehydrogenase; Mez: NAD- and NADP dependent malic enzymes; Pck: Phosphoenolpyruvate carboxykinase; Pgi: Phosphoglucose isomerase; Ppc: Phosphoenolpyruvate carboxylase; PTS: Phosphoenolpyruvate:carbohydrate phosphotransferase system; PykAF: Pyruvate kinases A and F; Zwf: Glucose 6-phosphate dehydrogenase; AcCoA: Acetyl-coenzyme A; CIT: Citrate; E4P: Erythose 4-phosphate; F6P: Fructose 6-phosphate; αKGT: α-ketoglutarate; GAP: Glyceraldehydes 3-phosphate; G6P: Glucose 6-phosphate; Glc: Glucose; GOx: Glyoxylate shunt; ICIT: Isocitrate; MAL: Malate; NADPH: Nicotinamide adenine dinucleotide phosphate (reduced form); NADH: Nicotinamide adenine dinucleotide (reduced form); OAA: Oxaloacetate; 2PG: 2-phosphoglycerate; 3PG: Phosphoglycerate; 6PGL: 6-phosphogluconolactone; PEP: Phosphoenolpyruvate; PYR: Pyruvate; R5P: Ribose 5-phosphate; SUCC: Succinate; BM: Biomass.

## Competing interests

The authors declare that they have no competing interests.

## Authors’ contributions

EM, FB, GG and CW participated in the design of this study. EM constructed the strains, realized the batch cultures and performed Pyk activity and HPLC determinations. JB designed the 1-^13^C glucose labeled experiments, the derivatization and GC-MS analysis of the hydrolizates. JB and CW did the modeling of the network for relative flux determination. EM, JB, FB, GG and CW participated in the analysis of the results as well as in writing and critical review of the manuscript. All authors have read and approved the manuscript.
